# Kidney transplant biopsy adequacy and outcomes in children – is it time for change?

**DOI:** 10.1186/s12882-025-04581-7

**Published:** 2025-12-03

**Authors:** Sarah Marokakis, Chen Cui, Ali Moghimi, Nicole Graf, Finbarr Coughlan, Siah Kim, Anne M. Durkan

**Affiliations:** 1 Department of Nephrology, The Children’s Hospital at Westmead, Cnr Hawkesbury Rd and Hainsworth St, Westmead, NSW 2145 Australia; 2 Department of Anaesthesia, St Vincent’s Hospital, Melbourne, Victoria, Australia; 3 Department of Histopathology, The Children’s Hospital at Westmead, Westmead, NSW Australia; 4 Faculty of Medicine and Health, The Children’s Hospital at Westmead Clinical School, University of Sydney, Sydney, NSW Australia; 5 Royal Brisbane and Women’s Hospital, Herston, Queensland Australia; 6 Queensland Children’s Hospital, South Brisbane, Queensland Australia; 7 Centre for Kidney Research, The Children’s Hospital at Westmead, Westmead, NSW Australia

**Keywords:** Transplant, Percutaneous kidney biopsy, Paediatrics

## Abstract

**Background:**

Percutaneous kidney transplant biopsies contribute to the diagnosis of transplant dysfunction and obtaining adequate tissue is crucial. Studies comparing biopsy outcomes according to technique are limited in the paediatric population and report variable outcomes. The aim of this study was to compare the adequacy and complication rates of kidney transplant biopsies performed using the tangential approach to those using the perpendicular approach.

**Methods:**

This was a retrospective, single-centre cohort study. Clinical, demographic and procedural data were analysed from the electronic medical records of kidney transplant recipients undergoing an allograft biopsy between January 2008 and December 2023. The Banff Classification of Renal Allograft Pathology was used to define sample adequacy.

**Results:**

Of 186 included biopsies, 100 (53.8%) were performed by IR and 86 (46.2%) by PN. The overall adequacy rate was 71.5% with a significantly higher adequacy rate in IR-performed biopsies (IR = 90% vs PN = 50%, *p* < 0.01). IR-performed biopsies had a significantly higher total glomeruli count (26 vs 12 glomeruli, *p* < 0.01) and glomeruli per core count (10 vs 7 glomeruli per core, *p* < 0.01). IR-performed biopsies had a significantly higher rate of obtaining one or more arteries (94% vs 69.8%, *p* < 0.01). There was no significant difference in complication rates between the PN and IR groups.

**Conclusions:**

There was an overall adequacy rate of 71% with significantly higher adequacy in IR-performed biopsies using a tangential approach. There were low rates of complications with no significant difference in rates between proceduralists.

## Background

Percutaneous kidney biopsy is key in the diagnosis of many kidney diseases in paediatrics. In the setting of kidney transplantation, biopsies are primarily utilised for the surveillance and detection of transplant rejection or other causes of transplant dysfunction [1]. Given the relatively poor correlation between clinical indicators and histopathological diagnosis [2], obtaining adequate biopsy samples for diagnosis is essential in guiding management in transplant recipients.

The Banff Classification of Allograft Pathology was established in 1993 following the first consensus meeting in 1991 [3]. It has since provided a framework for reporting kidney transplant biopsies including definitions of sample adequacy. The most recent update to the classification was in 2022, however the criteria for sample adequacy have been unchanged since 1997. In the paediatric population, obtaining adequate biopsy samples is achievable with one centre reporting a minimal or adequate sample in 62% of paediatric biopsies, a rate which increased if more than one core was collected [4].

**Fig. 1:**
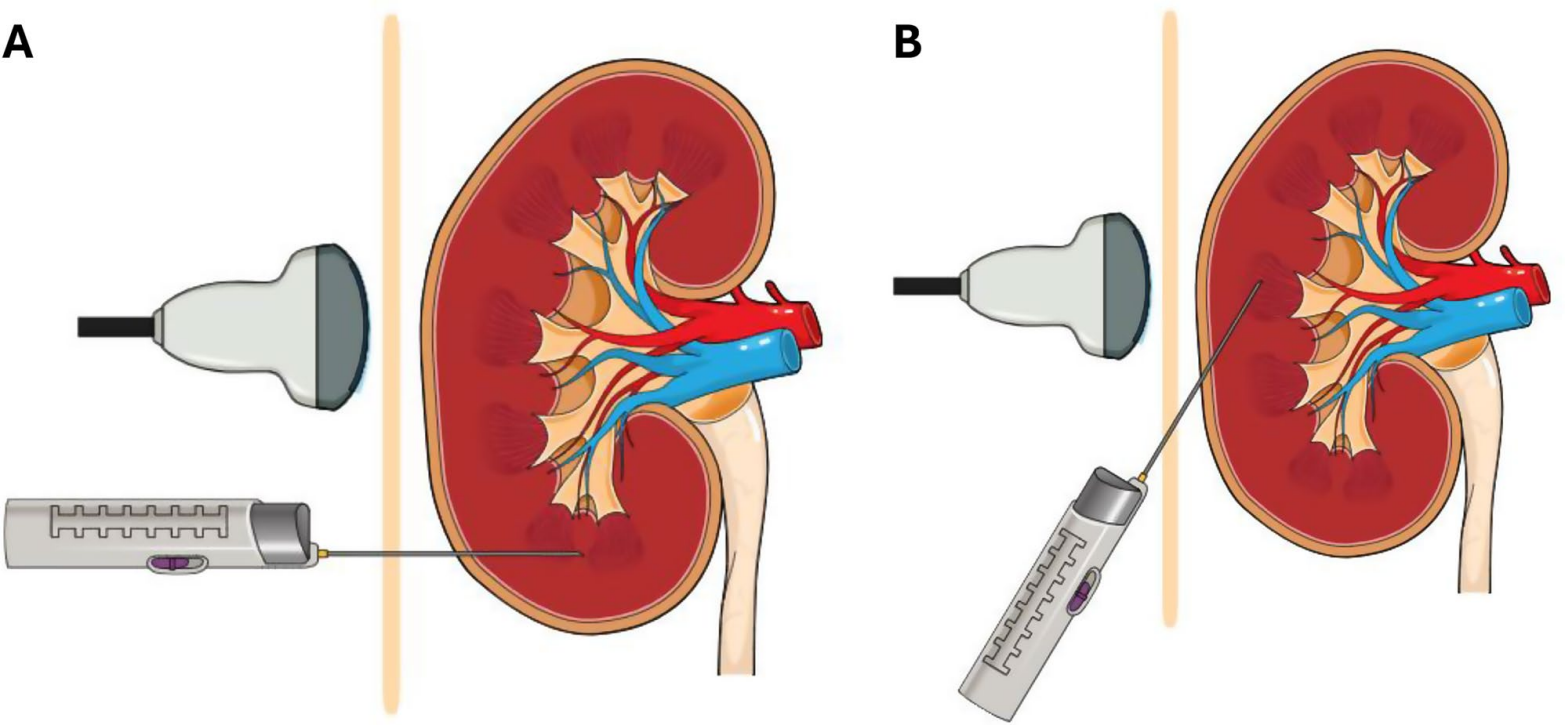
Comparison of percutaneous kidney biopsy techniques using the traditional perpendicular approach (Panel A) and tangential approach (Panel B)

With the evolution of biopsy techniques and changes to procedural practices over time [1], determining the most successful approach to transplant biopsies is important. Interventional radiologists (IR) are increasingly performing biopsies in place of paediatric nephrologists (PN) [5–7], which often allows access to elective procedure lists with anaesthesia support for delivery of general anaesthesia or sedation. This is likely leading to a decline in the procedural skills paediatric nephrologists and trainees, and further reducing the interventional aspect of the specialty. There has been an increased use of ultrasound-guided biopsy compared to ultrasound- localised biopsy where the site of entry is determined using ultrasonography however the biopsy is performed without real-time imaging [1, 6]. There have also been changes to the angle of approach of the biopsy needle to the cortex from a traditional perpendicular angle to a tangential approach [1, 8–11]. In the tangential approach (see Fig. 1), the biopsy needle approaches the cortex at a 45 degree angle under ultrasound guidance or localisation, with an anticipated increase in biopsy yield as the needle passes through a longer segment of cortex, capturing more glomeruli.

Studies comparing biopsy outcomes according to technique or proceduralist are lacking in the paediatric transplant population. One study that examined paediatric transplant biopsy yield between radiologists and nephrologists found significantly more glomeruli were obtained by radiologists [5]. However, it did not use the globally accepted Banff criteria to determine adequacy, instead defining a positive yield as one that provided ‘clinically important’ information. In contrast, another study found no significant difference in glomeruli yield in transplant biopsies between nephrologists and radiologists [12]. The tangential approach has been examined in native kidney biopsies in children with high rates of samples deemed adequate for diagnosis [10, 11]. There is also evidence that whilst overall adequacy may not vary between the tangential approach of IR and perpendicular approach of nephrologist, there is an increase in glomeruli obtained in IR-performed biopsy samples [7]. In the adult literature, comparisons between nephrologist and interventional radiologists had variable results in terms of adequacy and complication rates [6, 12–16]. There is a reported overall adequacy rate range of 85–93.8% in adult transplant biopsies [6, 8, 9]. However, whilst Chung et al. found a significantly higher glomeruli yield in radiology-performed biopsies, with a mean of 29 compared to a mean of 20 in nephrologist-performed biopsies [14], other studies found no significant difference in overall adequacy [6] or glomeruli yield between these groups [15]. With the exception of Tondel et al., which obtained multicentre data from a national registry [13], all the current literature in this area is from single centre studies.

The aims of this study were to compare the adequacy and complication rates of kidney transplant biopsy samples between interventional radiologists utilising the tangential approach and nephrologists using the perpendicular approach.

## Methods

This retrospective cohort study was performed at The Children’s Hospital at Westmead, Sydney, Australia, a large tertiary paediatric hospital with a kidney transplant unit. Ethical approval was granted by Sydney Children’s Hospitals Network Human Research Ethics Committee (SCHN HREC, HREC approval: LNR/17/SCHN/474) which subscribes to the ethical standards outlined in the Declaration of Helsinki. This ethics approval included a waiver of participant consent.

A search of the histopathology database was used to identify all kidney transplant allograft biopsies over a 16-year period between January 2008 and December 2023. Biopsies were included if performed in a child 18 years old or less by an interventional radiologist (IR), or paediatric nephrologist (PN) including consultants or trainees under supervision by a consultant. Back table biopsies at the time of transplantation and intra-operative biopsies performed by a surgeon at any time after initial transplantation were excluded. Our centre does not perform protocol biopsies; therefore all biopsies were ‘for-cause’ with any indication for biopsy included in this study.

### Biopsy technique

Biopsies performed by the IR were conducted in operating theatres or the interventional radiology suite, under general anaesthesia (GA). All biopsies were guided by real-time ultrasound performed by the IR performing the biopsy. The radiologist used a tangential approach at 45 degrees to the kidney. An 18-gauge biopsy needle was used in all biopsies using either a Bard Mission biopsy gun (Bard Biopsy, Tempe, Arizona, USA) with a coaxial system or a Biopince Full Core biopsy gun (Argon Medical Devices, Athens, Texas, USA).

Biopsies performed by PN were conducted in a procedure room in the outpatient clinic or ultrasound department under sedation usually consisting of oral morphine, oral midazolam and inhaled nitrous oxide. An ultrasonographer used ultrasound to provide real-time guidance during the procedure. The nephrologist used a perpendicular approach to the kidney. An 18-gauge biopsy needle was used in all biopsies using a Bard Max Core biopsy gun (Bard Biopsy, Tempe, Arizona, USA).

The decision regarding which proceduralist conducted the biopsy was dependent on availability of either the IR or PN list, and an assessment of complexity and co-morbidities of the patient with those deemed more complex or those with contraindications to sedation being referred to IR. Furthermore, due to a change in staffing resources at the centre, most biopsies from 2018 onwards were performed by IR.

All core samples were immediately examined by a histopathologist under a dissecting microscope to assess for the presence of glomeruli. When requested, this initial assessment was completed at the time of biopsy to allow further cores to be collected if tissue was inadequate, however this was not routine practice. After standard tissue fixation, processing, sectioning and staining (including three slides stained by haematoxylin and eosin, and three slides stained by periodic Schiff-methenamine, trichrome and periodic acid Schiff), the biopsies were examined by at least one histopathologist under light microscopy. If requested by the referring nephrologist, core samples may have also been sent for electron microscopy and/or immunofluorescence.

Following the procedure, the child was observed for a minimum of 6 hours on bedrest for any complications including haematuria. If the patient was stable with no significant complications, and there was no other indication to remain in hospital, he or she was discharged.

### Data collection

Demographic and clinical data were collected from the electronic medical records for each patient. Details of the biopsy procedure were obtained from the operation or procedure report documented by the proceduralist. Histopathology reports were reviewed to assess the adequacy of the biopsy sample, including the number of cores, and the number of glomeruli and vessels sampled, as well as the pathological diagnosis. In accordance with the Banff Classification of Renal Allograft Pathology, a biopsy was defined as adequate if it contained at least 10 glomeruli and 2 arteries [3]. A minimal sample was defined as containing 7 glomeruli and 1 artery [3]. The diagnosis was reported in accordance with the diagnostic categories outlined in the Banff Classification. In cases of inadequate information in the report, biopsy samples were re-examined by a histopathologist.

Data on post-biopsy complications were collected, including new onset macroscopic haematuria within 48 hours of biopsy, biopsy-related pain requiring any analgesia, infection, perinephric haematoma defined as a perinephric collection of any volume within 48 hours of biopsy, arterio-venous fistula formation, and need for blood product transfusion.

### Statistical analysis

The analysis was completed in Jamovi 2.3.28. Results are expressed as percentages, median values with interquartile ranges if data was not normally distributed, and mean values with standard deviation for normally distributed data. Difference in continuous variables were analysed using the students t test for normally distributed variables and the Mann-Whitney *U* test for non-normally distributed variables. Difference in categorical variables were analysed using the Pearson’s chi-squared test. To adjust for repeated biopsies in patients, we used a logistic generalised estimating equation with an exchangeable correlation matrix when investigating the association between biopsy operator and adequacy. A *p* value of < 0.05 was considered statistically significant.

## Results

A total of 186 kidney transplant biopsies from 76 individuals were included with 100 biopsies performed by IR (53.8%) and 86 biopsies performed by PN (46.2%). There was a total of five independent proceduralists in the IR group, including, three who were both trainees and consultants during the study period and more than 10 independent proceduralists in the PN group including both consultants and advanced trainees/fellows. In the PN group, 31 biopsies (36%) were performed by consultants, 44 biopsies (51.2%) by advanced trainees/fellows, and in 11 biopsies (13%) the proceduralist position was undocumented. The status of the IR proceduralist was not always documented. The characteristics of patients at the time of biopsy are presented in Table 1. The age of individuals at the time of biopsy was significantly higher in the PN group compared to the IR group (median: 14.3 vs 11.8 years, *p* < 0.01). Patient weight was also significantly higher in the PN group (mean: 44.8 vs 39.3 kg, *p* = 0.03), however height (median: 147.3 vs 143.9 cm, *p* = 0.15) and BMI (20.2 vs 19.7 kg/m^2^, *p* = 0.03) were similar across the groups. There was no significant difference in the number of patients who were overweight (23 vs 29, *p* = 0.11) or obese (10 vs 11, *p* = 0.56) between the IR and PN groups.

**Table 1:** Patient characteristics at the time of biopsy comparing ir- and PN-performed biopsies

Characteristic	Interventional Radiologist (*n* = 100)	Paediatric Nephrologist (*n* = 86)	p value
Age (years), *median (IQR)*	11.8 (8.7–14.1)	14.3 (9.9–16.2)	0.002
Sex, *n (%)*			
Males	68 (68)	61 (70.9)	0.67
Height (cm), *median (IQR)*	143.9 (116.3–153.8)	147.3 (133.2–155.2)	0.15
Weight (kg), *mean (SD)*	39.3 (17.2)	44.8 (17.4)	0.03
Body Mass Index (BMI, kg/m^2^), *median (IQR)*	19.7 (17.5–22.2)	20.2 (18.1–24.2)	0.28
Overweight, *n (%)*	23 (23)	29 (33.7)	0.11
Obese, *n (%)*	10 (10)	11 (12.8)	0.56
Blood Pressure (mmHg), mean (SD)			
Systolic	115 (13)	119 (12)	0.02
Diastolic	70 (13)	74 (12)	0.03
Estimated Glomerular Filtration Rate (eGFR, ml/min/1.73 m^2^), *mean (SD)*	58 (22)	47 (25)	0.002
Urea (mmol/L), *median (IQR)*	8.5 (6.7–12)	10 (7.1–14.6)	0.05
Haemoglobin (g/L), *median (IQR)*	113 (99–120)	110 (99–121)	0.65
Platelets (x10^9^/L), *median (IQR)*	277 (223–346)	255 (213–311)	0.13
Coagulation			
Prothrombin Time (PT, sec), *median (IQR)*	11.8 (11–13.4)	13.5 (12–14.2)	< 0.001
INR, *median (IQR)*	1 (0.9–1)	1 (1–1.1)	0.13
Activated Partial Thromboplastin-clotting Time (aPTT, sec), *median (IQR)*	29.6 (27.9–32.6)	30.1 (27.4–32.3)	0.46

Baseline mean eGFR was significantly lower in the PN group (47 vs 58 ml/min/1.73 m^2^, *p* = 0.002). Comparison of baseline coagulation studies found a significantly longer prothrombin time in the PN group (12.5 vs 11.8 sec, *p* < 0.001), however similar INR (1 vs 1, *p* = 0.13) and aPTT (30.1 vs 29.6 sec, *p* = 0.46). Baseline haemoglobin and platelet counts were similar across the groups.

Table 2 outlines the indications for biopsy and histological diagnosis. The most common indication for biopsy was elevated creatinine (73.7%), followed by presence of donor specific antibodies (18.8%), and previous episode of rejection (17.7%). There was no significant difference in the indications for biopsy between PN and IR- performed biopsies.

**Table 2:** Comparison of indications for biopsy and histological diagnosis between IR- and PN-performed biopsies

	Interventional Radiologist (*n* = 100)	Paediatric Nephrologist (*n* = 86)	p value
Indication for Biopsy, *n (%)*			
Elevated creatinine	72 (72)	65 (76)	0.58
Follow up of rejection	22 (22)	11 (13)	0.1
Donor specific antibodies	22 (22)	13 (15)	0.23
Delayed graft function	3 (3)	5 (6)	0.35
Other	13 (13)	7 (8)	0.29
Histological Diagnosis, *n (%)*			
No rejection	56 (56)	53 (62)	0.44
Antibody-mediated rejection	19 (19)	7 (8)	0.03
T-cell mediated rejection	10 (10)	9 (11)	0.92
Borderline rejection	13 (13)	13 (15)	0.68
Mixed rejection	5 (5)	2 (2)	0.34
Chronic allograft nephropathy	10 (10)	8 (9)	0.87
Acute tubular necrosis	23 (23)	28 (33)	0.15
Other	33 (33)	19 (22)	0.1
No diagnosis possible	2 (2)	2 (2)	0.88

As shown in Table 3, the overall adequacy rate was 71.5%, with a significantly higher adequacy rate in the IR group compared to the PN group (90% vs 50%, *p* < 0.01). IR-performed biopsies had a significantly higher total glomeruli count (26 vs 12 glomeruli, *p* < 0.01) and glomeruli per core count (10 vs 7 glomeruli per core, *p* < 0.01). IR-performed biopsies had a significantly higher rate of obtaining one or more arteries (94% vs 69.8%, *p* < 0.01). Of those samples which did not meet adequacy criteria, 47.2% met the criteria for a minimal sample (IR = 30% vs PN = 52.2%, *p* = 0.23).

**Table 3:** Biopsy yield and adequacy comparing interventional radiologist and paediatric nephrologist performed biopsies

	Interventional Radiologist (*n* = 100)	Paediatric Nephrologist (*n* = 86)	p value
Cores, *median (IQR)*	3 (2–3)	2 (1–2)	< 0.001
Glomeruli, *median (IQR)*			
Total glomeruli	26 (16–40)	12 (7–18)	< 0.001
Glomeruli per core	10 (7–15)	7 (4–10)	< 0.001
Arteries, *n (%)*			
< 1	6 (6)	26 (30.2)	< 0.001
≥1	94 (94)	60 (69.8)	
Overall Adequacy, *n (%)*	90 (90)	43 (50)	< 0.001
Adequate glomeruli, *n (%)*	91 (91)	51 (59.3)	< 0.001
Adequate arteries, *n (%)*	94 (94)	61 (70.9)	< 0.001

Acute rejection was diagnosed in 78 biopsies (41.9%), with antibody-mediated (14%) and borderline T-cell mediated rejection (14%) being the most common rejection subtypes. Acute tubular necrosis was present in 51 biopsies (27.4%), chronic allograft nephropathy in 18 biopsies (9.7%), and other diagnoses in 52 biopsies (28%), including calcineurin toxicity, recurrence of primary disease, interstitial fibrosis or ischaemic changes. No diagnosis was possible in 4 biopsies (2.2%) due to inadequate samples, with 2 in the PN and 2 in the IR groups (*p* = 0.88).

A comparison of biopsy-related complications is outlined in Table 4. Post-biopsy pain requiring analgesia was the most common complication occurring in 58 cases (31.2%), followed by de novo macroscopic haematuria occurring in 21 cases (11.3%), and perinephric haematomas (*n* = 14, 7.5%). One patient required a blood transfusion post-biopsy. No biopsy-related infection or arteriovenous fistula formation was observed. There was no significant difference in complication rates between the PN and IR group.

**Table 4:** Comparison of biopsy-related complications following biopsies performed by interventional radiologist and paediatric nephrologists

	Interventional Radiologist (*n* = 100)	Paediatric Nephrologist (*n* = 86)	p value
Pain-requiring analgesia, *n (%)*	35 (35)	23 (26.7)	0.23
Macroscopic haematuria, *n (%)*	9 (9)	12 (14)	0.29
Perinephric haematoma, *n (%)*	7 (7)	7 (8.1)	0.77
Transfusion, *n (%)*	1 (1)	0 (0)	0.35
Biopsy-related infection, *n (%)*	0 (0)	0 (0)	
Arteriovenous fistula, *n (%)*	0 (0)	0 (0)	

The 186 biopsies included were performed in 76 individual patients with a range of 1 – 8 biopsies performed in each individual within the study period. In the analysis correcting for the potential effect of some individuals having multiple biopsies, there remained a significant difference in adequacy rates, with the odds of obtaining an adequate sample in the PN group being 89% less than the IR group (OR: 0.11, 95% CI: 0.06–0.24, *p* < 0.001). We also used this analysis to assess the effect of age and BMI on the outcomes. Adequacy rates dropped by 7% with every 1 year increase in age of the patient (OR: 0.93, 95% CI: 0.85–1.02). Adequacy rates were also reduced in overweight (OR: 0.8, 95% CI: 0.26–2.42) and obese (OR: 0.49, 95% CI: 0.13–1.86) individuals, however neither the effect of age or overweight/obesity were statistically significant (*p* = 0.13 and 0.58 respectively).

## Discussion

Percutaneous kidney transplant biopsies remain an important tool in the assessment of graft function. However, there has been little evidence examining the adequacy of transplant biopsies in children using different biopsy techniques that have emerged over time. In this study, we found an overall adequacy rate of 71.5% with a significantly higher adequacy rate in the IR-performed biopsies (90%) using the tangential approach compared to the paediatric nephrologist group (50%) using a perpendicular approach, with similar complication rates. This significant difference in adequacy rate may have important clinical implications where the diagnosis and management of graft dysfunction relies on obtaining an adequate sample for assessment.

In other paediatric transplant cohorts, adequacy rates have been variable. One study, of PN performed biopsies, found an adequacy rate of 35%, with a minimal sample able to be obtained in 62% of biopsies [4]. In comparison, Feins et al. found an adequacy rate of 82% in transplant biopsies in their cohort but they did not use the Banff adequacy criteria and instead a definition of ‘clinically important information’ [5]. Given that in less than 3% of our biopsies, no diagnosis was made, it could be argued that clinically important information was achieved in 97%. The studies in adult transplant patients found higher adequacy rates compared to our study, with a range of 81.6–95% [6, 8, 9, 17], but again there was some heterogeneity in the definition of adequate. The Banff criteria provide an approach to transplant biopsies which is standardised and aims to minimise subjectivity. Without consistency in the definition of an adequate sample used in research, it is difficult to make meaningful comparisons of outcomes across cohorts.

Interventional radiologists at our hospital use the tangential approach to the cortex in kidney biopsies compared to the traditional perpendicular approach used by nephrologists. In our study, we found that the IR-performed biopsies utilising this technique had a significantly higher adequacy rate of 90% compared to nephrologist-performed biopsies which had an adequacy rate of only 50%. The tangential approach allows the biopsy needle to traverse a longer path through the cortex of the kidney and therefore would be expected to obtain a larger cortical sample with more glomeruli and small artery sections included in the core. The tangential approach in transplant biopsies in adults has been found to have high adequacy rates of 86.7 and 95% [8, 9], however neither study had a comparison group to assess if it increased biopsy yield compared to a perpendicular approach. Comparison of techniques in the paediatric population has focussed on native kidney biopsies with variable results. Cakmakci et al. found only one case in 166 patients had a non-diagnostic sample due to inadequate material using a tangential approach [10]. Caliskan et al. found biopsies using the tangential approach had a significantly increased adequacy rate compared to perpendicular approach [11], whereas Pettit et al. found that whilst the glomeruli yield was higher in IR-performed biopsies using a tangential approach, the overall adequacy rate was not significantly higher than PN-performed perpendicular biopsies [7]. One potential confounder in our study was the higher use of general anaesthesia by IR, potentially facilitating the procurement of tissue in some children.

When considering the effect of proceduralist rather than technique, our study found contrasting results to the current evidence. Using heterogeneous definitions, several studies found no difference in adequacy [5, 7, 16]. The effect of operator on glomeruli count is variable with some studies finding higher counts, whereas others found lower counts [5, 7, 16].

Furthermore, whilst some studies found no difference in glomeruli counts according to proceduralist [12, 15], others found IR had lower glomeruli yield compared to nephrologists [6, 14]. In our centre, there is a larger number of independent proceduralists in the PN group compared to the IR group, as well as a larger number of trainees in the PN group. There is a possibility that due to the larger number of independent proceduralists in the PN group, the relative procedural experience is lower in the PN group compared to IR group which may impact on technique and biopsy yield.

The impact of several technical factors on biopsy yield have been investigated, including biopsy needle size, number of cores, ultrasound technique and use of microscopic evaluation at the time of biopsy. In terms of biopsy needle size, several studies found no difference in biopsy yield according to needle size [4, 6, 13, 18], though Sekulic et al. found IR-performed biopsies using a larger needle had increased glomeruli counts and were less likely to have an insufficient diagnosis [16]. Complication rates were not compared in the Sekulic study [16], however Tondel et al. found an increase rate of haematoma formation with a larger needle size [13], therefore a balance of risk and benefit needs to be considered. In our study, all biopsies were performed with an 18 g needle. There were variations in the number of cores collected by proceduralists, and we postulated that the increased number of cores would increase biopsy yield given the larger amount of tissue collected. To correct for this, we compared the number of glomeruli per core in each group and found the IR-performed biopsies still had a significantly higher average compared to PN-performed biopsies.

We were unable to determine the effect of immediate microscopic evaluation on outcomes as this was not documented in the medical record, however there is evidence that the use of microscopic evaluation at the time of biopsy increased glomerular yield even if a smaller core was obtained [16, 18].

The ultrasound technique used varied in our study, with IR and some nephrologists using real-time ultrasound guidance, whilst other nephrologists used ultrasound-marked or localised technique. The technique was not consistently recorded in the medical record therefore we were unable to perform any analysis to assess the impact of this. Several studies have compared different ultrasound techniques in native and transplant biopsies and have found no difference in biopsy yield between ultrasound-guided or ultrasound localised biopsies [6, 14, 19]. Furthermore, Sekulic et al. found no difference in biopsy adequacy comparing ultrasound and CT-guided biopsy [16]. This suggests that imaging technique may not significantly influence biopsy adequacy.

We found that biopsy adequacy decreased with increasing age which was unexpected. We would anticipate the biopsy to be easier in older children both due to compliance with sedation and their body size, however this was not reflected in the results. It is possible there are other confounding factors contributing to this which have not been identified in this study due to its retrospective design.

The complications rates in our cohort were small with no significant difference according to biopsy technique used. The tangential approach has been found to be a safe technique with low major complications rates of less than 2% in adult studies [8, 9], and a significant reduction in complication rates in paediatric native kidney biopsies of 9.6% in the tangential approach compared to 20.3% in the perpendicular approach [11]. However, comparison of complication rates is more variable when examining the impact of the proceduralist. Most studies have found no difference in complication rates or severity between radiology- and nephrology performed biopsies [6, 12–14]. However Pettit et al. found there to be an increase in reported haematoma formation in paediatric nephrology-performed native kidney biopsies [7], whilst Gupta et al. found a significant reduction in severe complications in adult native kidney biopsies performed by nephrologists [15]. There are reported differences in complications rates between adult and paediatric populations which may explain these different findings [13]. Regardless of the technique or population sample, haematomas and macroscopic haematuria are the most common complications which is also reflected in our results.

There were several limitations in this study. It is a retrospective single-centre study which may limit generalisability of the study. There were differences in the baseline patient characteristics between the groups, particularly age and weight. As discussed, there may be other characteristics that influenced the adequacy rates that we were unable to control with the study design, including the use of general anaesthesia in the IR group compared to sedation in the nephrologist group, or the use of proceduralist operated ultrasound guidance compared to the ultrasound-localised technique. Furthermore, there have been multiple proceduralists conducting kidney biopsies over the years in the study with the possibility of variable levels of experience and variations in techniques used in the sampled population, as well as differences in overall procedural experience between IR and PN. Due to the retrospective nature of this study, we did not assess patient-reported outcomes, therefore patient preferences and satisfaction with the procedure technique or outcomes could not be assessed.

## Conclusions

This study found a significantly higher proportion of adequate kidney transplant biopsies performed by IR using general anaesthesia and the tangential approach with low complication rates compared to the perpendicular approach performed by PN. Acknowledging the limitations of the retrospective study design, the significantly better outcome in the IR group would support consideration of adopting the tangential approach to maximise the yield and clinical utility of transplant biopsies in children.

## Data Availability

The datasets used and/or analysed during the current study are available from the corresponding author on reasonable request.

## Abbreviations

eGFR: Estimated glomerular filtration rate
GA: General anaesthesia
IR: Interventional nephrologist
PN: Paediatric nephrologist
